# The Impacts of Using Smartphone Dating Applications on Sexual Risk Behaviours in College Students in Hong Kong

**DOI:** 10.1371/journal.pone.0165394

**Published:** 2016-11-09

**Authors:** Edmond Pui-Hang Choi, Janet Yuen-Ha Wong, Herman Hay-Ming Lo, Wendy Wong, Jasmine Hin-Man Chio, Daniel Yee-Tak Fong

**Affiliations:** 1 School of Nursing, the University of Hong Kong, Hong Kong S.A.R., People’s Republic of China; 2 Department of Applied Social Sciences, The Polytechnic University of Hong Kong, Hong Kong S.A.R., People’s Republic of China; 3 Hong Kong Institute of Integrative Medicine and School of Chinese Medicine, the Chinese University of Hong Kong, Hong Kong S.A.R., People’s Republic of China; 4 Department of Counselling & Psychology, Hong Kong Shue Yan University, Hong Kong S.A.R., People’s Republic of China; Agencia de Salut Publica de Barcelona, SPAIN

## Abstract

Dating applications (apps) on smartphones have become increasingly popular. The aim of this study was to explore the association between the use of dating apps and risky sexual behaviours. Data were collected in four university campuses in Hong Kong. Subjects completed a structured questionnaire asking about the use of dating apps, sexual behaviours, and sociodemographics. Multiple linear and logistics regressions were used to explore factors associated with sexual risk behaviours. Six hundred sixty-six subjects were included in the data analysis. Factors associated with having unprotected sexual intercourse with more lifetime sexual partners included use of dating apps (β = 0.93, p<0.01), having one’s first sexual intercourse before 16 years of age (β = 1.74, p<0.01), being older (β = 0.4, p<0.01), currently being in a relationship (= 0.69, p<0.05), having a monthly income at least HKD$5,000 (β = 1.34, p<0.01), being a current smoker (β = 1.52, p<0.01), and being a current drinker (β = 0.7, p<0.01). The results of a multiple logistic regression analysis found that users of dating apps (adjust odds ratio: 0.52, p<0.05) and current drinkers (adjust odds ratio: 0.40, p<0.01) were less likely to have consistent condom use. Users of dating apps (adjust odds ratio: 1.93, p<0.05), bisexual/homosexual subjects (adjust odds ratio: 2.57, p<0.01) and female subjects (adjust odds ratio: 2.00, p<0.05) were more likely not to have used condoms the last time they had sexual intercourse. The present study found a robust association between using dating apps and sexual risk behaviours, suggesting that app users had greater sexual risks. Interventions that can target app users so that they can stay safe when seeking sexual partners through dating apps should be developed.

## Introduction

The Internet has long been a popular platform for seeking romantic and even sexual relationships [1]. A large study (n = 7,037) in the United States found that 9.8% of respondents used the Internet to meet sexual partners [2]. Another study in Sweden found that 35% of men and 40% of women reported having had sex with a person met online [3]. A review article concluded that online sex seeking was associated with adverse sexual health such as sexually transmitted infections (STIs), unsafe sex and unplanned pregnancies in both heterosexual and homosexual populations, hence, it was suggested that people who seek sexual partners on the Internet tend to be more sexually active and more willing to take risks [4].

The advancement of mobile technology allows Internet access through smartphones. Along with the growing popularity of smartphones and Internet access worldwide, a variety of location-based dating applications (apps) using the global positional system (GPS) have been launched. Compared with traditional online dating paradigms such as websites and chat rooms, these dating apps provide a more convenient and accessible way for people to meet friends and potential sexual partners. First, users can easily find sexual partners who are geographically nearby because of the GPS. Second, nowadays people tend to carry their smartphones with them at all times. Users can easily access a wide pool of potential sexual partners anytime and anywhere as long as they carry their mobile phones. Third, unlike traditional dating websites which might require subscription fees, most of the dating apps are free. Therefore, it is highly possible that using dating apps is an emerging risk factor for unsafe sexual behaviours. Previous studies found that the prevalence of having unprotected anal intercourse ranged from 17.0% [5] to 66.7% [6] in homosexual men who used dating apps. Moreover, homosexual men who used dating apps for more than one year were more likely to have unprotected anal intercourse [7]. Compared with nonusers, app users were significantly more likely to have at least one self-reported prior diagnosis of STIs [8]. Insight into the use of social dating apps and its associated risky sexual behaviours will both allow researchers and clinicians to understand the phenomenon and guide the design of tailored interventions.

However, to date, the evidence regarding the negative effects of smartphone dating apps on sexual health has been conflicting, inconclusive or not generalizable. A study in the United States found that homosexual app users were more likely to have more sexual partners [8] whereas another study in Hong Kong did not find this association [9]. Furthermore, previous studies mainly focused on homosexual men. The association between using dating apps and sexual health in heterosexual people and women is poorly understood. Distinct differences in sexual practices and sexual risks between people of different genders [10] and sexual orientations [11] have been evident in previous studies. For example, men were more likely to intend to engage in sexual activities than women [10]. Oral and anal sexual intercourse was more common in homosexual men then heterosexual men. Bisexual and homosexual women were more likely to have more sexual partners than heterosexual women [11]. The lack of knowledge about the effects of using dating apps on sexual health in male and female subjects of various sexual orientations necessitated the present study.

The specific objective of the present study was to explore the association between the use of smartphone dating apps and risky sexual behaviours, including the number of sexual partners with whom students have had unprotected sexual intercourse, inconsistent condom use and not using a condom the last time students had had sexual intercourse. It was hypothesized that the use of dating apps was associated with these behaviours.

## Methods

### Subjects and sampling

This was a cross-sectional study. Subjects were recruited from four university campuses in Hong Kong by convenience sampling. Subjects were excluded if they did not speak or understand English, Cantonese or Mandarin; had already been recruited to the study; or were not college students.

A coded and anonymous questionnaire was self-completed by subjects. Questionnaires were available in English and Chinese. Bilingual field workers were present to explain the aims, procedures and nature of the study; obtain written consent; distribute and collect questionnaires; and answer questions raised by subjects. Subjects were informed that they could skip any questions they did not want to answer.

### Study instruments

The questions about the use of dating apps, sexual behaviours and sexual orientation were adopted from previous studies [5, 8, 12–17].

#### Use of dating apps

Subjects were asked if they were using any smartphone dating apps[5]. Users of dating apps were asked how long they had been using them [5, 16]. The operational definition of dating app was that a smartphone’s application was primarily for dating; that it used GPS technology, in which users can locate other users nearby; and that users could send text messages and exchange pictures [5, 12], for example, Tinder, Skout, Grindr and Jack’d. Messenger apps that were primarily developed for communication but also have a secondary function to look for “new friends nearby” were not considered in this present study.

#### Sexual behaviours

Subjects were asked if they had ever had sexual intercourse [15]. Those who had sexual intercourse experience were asked at what age they had had their first sexual intercourse[15]; the gender of their sexual partners [17]; the number of sexual partners in the past 1 month, the past 3 months and their entire lives [8, 13, 15]; the number of sexual partners they had had unprotected sexual intercourse with; the frequency of their condom use[9]; and whether they had used a condom the last time they had had sexual intercourse [15].

#### Sociodemographics

All subjects also completed a set of sociodemographic questions about age, gender, sexual orientation, relationship status, monthly income, student status, smoking habits and drinking habits.

The study instrument is shown in S1 Instrument.

### Sample size calculation

A previous study found the effect size (Cohen’s d) of difference in unprotected sexual intercourse between young adults who had sexted (sent or received sexually explicit photos on mobile phones) and those who did not was 0.35 [18]. Using this as a reference point, it was calculated that a minimum sample size of 67 subjects was needed to detect a difference by independent t-test with a power of 95% and a two-tailed significance of 0.05.

### Statistical analysis

Fisher’s exact test and multiple logistic regression analyses were conducted to identify factors associated with having sexual intercourse experience, having consistent condom use and not having used a condom the last time one has had sexual intercourse. Multiple linear regression analyses were conducted to identify factors associated with more sexual partners in one’s lifetime, the past 3 months and the past 1 month, and having had unprotected sex with more lifetime sexual partners. Multicollinearity diagnostics were performed for all regression models. In each regression model, only subjects with full data were included in the analysis. Imputation or other substitution methods were not used.

All statistical analyses were conducted by the SPSS 23 with *p* values < 0.05 indicating statistical significance.

### Ethics

All authors declare they have no conflict of interest.

Ethics approval was obtained from the HKU/HA HKW institutional review board, the Joint CUHK-NTEC Clinical Research Ethics Committee and City University of Hong Kong Human Subjects Ethics Sub-Committee.

The methods were carried out in accordance with the approved guidelines. Written informed consent was obtained from all individual participants included in the study.

All procedures performed in studies involving human participants were in accordance with the ethical standards of the institutional and/or national research committee and with the 1964 Helsinki declaration and its later amendments or comparable ethical standards.

## Results

### Subjects characteristics

Subjects were recruited between September 2015 and December 2015. A total of 676 subjects completed the cross-sectional survey. Of these, 666 subjects were included in the data analysis; the other 10 subjects were excluded because they indicated that they were not college students. The mean age was 20 years; 54.1% were female, 82.4% were heterosexual, 71.5% were currently in a dating relationship, 60.5% did not have sexual intercourse experience and 52.9% currently used dating apps. Demographic information is shown in Table 1.

**Table 1:** Demographic information of participants.

		Overall
		n = 666
**Demographics**		
Mean age (SD)		20.03 (1.52)
Gender, n (%)		
	Male	296 (44.44)
	Female	360 (54.05)
	Did not answer	10 (1.50)
Sexual orientation, n (%)		
	Heterosexual	549 (82.43)
	Bisexual/homosexual	113 (16.97)
	Did not answer	4 (0.60)
Relationship status, n (%)		
	Not currently in a relationship	190 (28.53)
	Currently in a relationship	476 (71.47)
Monthly income, n (%)		
	<HKD5,000	525 (78.83)
	≥HKD 5,000	127 (19.07)
	Did not answer	14 (2.10)
Smoking status, n (%)		
	Non current smoker	636 (95.50)
	Current smoker	28 (4.20)
	Did not answer	2 (0.30)
Drinking status, n (%)		
	Non current drinker	359 (53.90)
	Current drinker	305 (45.80)
	Did not answer	2 (0.30)
Sexual intercourse, n (%)		
	No	403 (60.51)
	Yes	260 (39.04)
	Did not answer	3 (0.45)
Use of Dating apps		
	Yes	352 (52.85)
	No	312 (46.85)
	Did not answer	2 (0.30)

### Factors associated with having sexual intercourse experience

Factors associated with having sexual intercourse experience, the corresponding chi-square statistics, adjusted odds ratios (aOR) derived from multiple logistic regression analyses and model evaluation statistics are shown in Table 2. All factors that were significant in the chi-square analysis remained statistically significant in the multiple logistic regression analysis except for gender and monthly income. Factors associated with having sexual intercourse experience included using dating apps (aOR: 1.87), being older (aOR: 1.39), being bisexual or homosexual (aOR: 4.14), currently being in a dating relationship (aOR: 2.14), being a current smoker (aOR: 6.14) and being a current drinker (aOR: 2.68).

**Table 2:** Correlates of having sexual intercourse experience.

		Sexual intercourse experience		p-value^	Multiple logistic regression (n = 638)		
		Yes	No		Adjusted OR (95% CI)		p-value
**Use of dating apps, n (%)**				p<0.01			p<0.01
	No	90 (34.62)	222 (55.09)		1.00		
	Yes	170 (65.38)	181 (44.91)		**1.87 (1.29–2.72)**		
**Mean age (SD)**				p<0.01			p<0.01
		20.54 (1.66)	19.71 (1.33)		**1.39 (1.22–1.59)**		
**Gender, n (%)**				p<0.01			0.33
	Male	134 (52.34)	161 (40.45)		1.00		
	Female	122 (47.66)	237 (59.55)		0.83 (0.57–1.21)		
**Sexual orientation, n (%)**				p<0.01			p<0.01
	Heterosexual	180 (69.77)	367 (91.29)		1.00		
	Bisexual/homosexual	78 (30.23)	35 (8.71)		**4.14 (2.50–6.84)**		
**Relationship status, n (%)**				p<0.05			p<0.01
	Not currently in a relationship	62 (23.85)	127 (31.51)		1.00		
	Currently in a relationship	198 (76.15)	276 (68.49)		**2.14 (1.38–3.30)**		
**Monthly income, n (%)**				p<0.01			0.16
	<HKD5,000	184 (72.73)	339 (85.39)		1.00		
	≥HKD 5,000	69 (27.27)	58 (14.61)		1.41 (0.87–2.29)		
**Smoking habit, n (%)**				p<0.01			p<0.01
	Non- or ex-smoker	234 (90.35)	399 (99.25)		1.00		
	Current smoker	25 (9.65)	3 (0.75)		**6.14 (1.69–22.34)**		
**Drinking habit, n (%)**				p<0.01			p<0.01
	Non- or ex- drinker	98 (37.84)	259 (64.43)		1.00		
	Current drinker	161 (62.16)	143 (35.57)		**2.68 (1.84–3.91)**		** **

All variance inflation factors < 2.

^ p-value by Fisher’s exact test for categorical variables and independent t-test for continuous variables

### Factors associated with the number of sexual partners

Two hundred sixty subjects who had sexual intercourse experience were further analysed to explore factors associated with their sexual behaviours. Their demographic information is shown in Table 3.

**Table 3:** Demographic information and sexual behaviours of participants who have sexual intercourse experience.

		Overall	App users	Non app users
		n = 260	n = 170	n = 90
**Demographics**				
Mean age (SD)*		20.54 (1.66)	20.75 (1.70)	20.15 (1.52)
Gender, n (%)^				
	Male	134 (51.54)	98 (57.65)	36 (40.00)
	Female	122 (46.92)	71 (41.76)	51 (56.67)
	Did not answer	4 (1.54)	1 (0.59)	3 (3.33)
Sexual orientation, n (%)^^				
	Heterosexual	180 (69.23)	105 (61.76)	75 (83.33)
	Bisexual/homosexual	78 (30.00)	64 (37.65)	14 (15.56)
	Did not answer	2 (0.77)	1 (0.59)	1 (1.11)
Relationship status, n (%)				
	Not currently in a relationship	62 (23.85)	46 (27.06)	16 (17.78)
	Currently in a relationship	198 (76.15)	124 (72.94)	74 (82.22)
Monthly income, n (%)^^				
	<HKD5,000	184 (70.77)	110 (64.71)	74 (82.22)
	≥HKD 5,000	69 (26.54)	55 (32.35)	14 (15.56)
	Did not answer	7 (2.69)	5 (2.94)	2 (2.22)
Smoking status, n (%)^				
	Non current smoker	234 (90.00)	148 (87.06)	86 (95.56)
	Current smoker	25 (9.62)	21 (12.35)	4 (4.44)
	Did not answer	1 (0.38)	1 (0.59)	0 (0.00)
Drinking status, n (%)				
	Non current drinker	98 (37.69)	60 (35.29)	38 (42.22)
	Current drinker	161 (61.92)	109 (64.12)	52 (57.78)
	Did not answer	1 (0.38)	1 (0.59)	0 (0.00)
**Sexual behaviours**				
Number of sexual partners (lifetime)**		3.26 (3.62)	4.07 (3.82)	1.72 (2.59)
Number of sexual partners (3 months)**		1.01 (0.69)	1.12 (0.73)	0.81 (0.56)
Number of sexual partners (1 months)*		0.75 (0.57)	0.80 (0.60)	0.65 (0.50)
Number of sexual partners (unprotected sexual intercourse, lifetime) **		2.00 (2.55)	2.63 (2.79)	0.80 (1.40)
Age of the first sexual intercourse^^				
	<16 years	37 (14.23)	32 (18.82)	5 (5.56)
	≥16 years old	223 (85.77)	138 (81.18)	85 (94.44)
Consistent condom use^^				
	Inconsistent	186 (71.54)	134 (78.82)	52 (57.78)
	Consistent (100%)	74 (28.46)	36 (21.18)	38 (42.22)
Type of sexual partners (last time they had sexual intercourse) ^^				
	Committed partner (boyfriend/girlfriend)	205 (78.85)	121 (71.18)	84 (93.33)
	Casual partner	55 (21.15)	49 (28.82)	6 (6.67)
Condom use (last time they had sexual intercourse) ^^				
	No	125 (48.08)	97 (57.06)	28 (31.11)
	Yes	133 (51.15)	72 (42.35)	61 (67.78)
	Did not answer	2 (0.77)	1 (0.59)	1 (1.11)

*p-value<0.05 by independent t-test

**p-value<0.01 by independent t-test

^p-value<0.05 by Fisher’s exact test

^^p-value<0.01 by Fisher’s exact test

The results of multiple linear regression analysis found that factors associated with more lifetime sexual partners were being a user of dating apps, reporting first sexual intercourse before 16 years of age (the age of consent in Hong Kong), being older, having a monthly income greater than HKD$5,000 and being a current smoker. Factors associated with more sexual partners in the past 3 months were being a user of dating apps, currently being in a dating relationship and having a monthly income greater than HKD$5,000. Factor associated with a high number of sexual partners in the past 1 month were being currently in a dating relationship and having a monthly income greater than HKD$5,000 only. The results are shown in Table 4.

**Table 4:** Factors associated with the number of sexual partners by multiple linear regressions

**Independent variable**	**Number of lifetime sexual partners (n = 244)**				**Number of sexual partners in tde last 3 montds (n = 244)**			
	**Coeff**	**SE**	**95% CI**	**p-value**	**Coeff**	**SE**	**95% CI**	**p-value**
Use of dating apps, Yes (vs. No)	1.28	0.41	(0.48, 2.09)	p<0.01	0.23	0.09	(0.06, 0.41)	p<0.05
Age of the first sexual intercourse, ≥16 years old (vs. <16 years old)	-1.77	0.58	(-2.92, -0.63)	p<0.01	0.04	0.13	(-0.22, 0.29)	0.79
Age	0.35	0.12	(0.12, 0.59)	p<0.01	0.03	0.03	(-0.02, 0.09)	0.20
Gender, Female (vs. Male)	-0.47	0.39	(-1.24, 0.30)	0.23	-0.05	0.09	(-0.22, 0.12)	0.56
Sexual orientation, Bisexual/Homosexual (vs. Heterosexual)	-0.25	0.42	(-1.08, 0.57)	0.54	0.09	0.09	(-0.09, 0.28)	0.32
Relationship status, Currently in a relationship (vs. Not currently in a relationship)	0.26	0.45	(-0.63, 1.14)	0.57	0.42	0.10	(0.22, 0.61)	p<0.01
Monthly income, ≥HKD 5,000 (vs. <HKD5,000)	2.19	0.46	(1.29, 3.10)	p<0.01	0.25	0.10	(0.05, 0.45)	p<0.05
Smoking status, Current smoker (vs. Non current smoker)	3.43	0.66	(2.14, 4.73)	p<0.01	0.18	0.15	(-0.10, 0.47)	0.21
Drinking status, Current drinker (vs. Non current drinker)	0.55	0.40	(-0.24, 1.34)	0.17	0.04	0.09	(-0.14, 0.21)	0.67
R-square	41.54%				15.84%			
**Independent variable**	**Number of sexual partners in the last 1 month (n = 244)**				**Number of lifetime sexual partners with unprotected sexual intercourse (n = 242)**			
** **	Coeff	SE	95% CI	p-value	Coeff	SE	95% CI	p-value
Use of dating apps, Yes (vs. No)	0.10	0.07	(-0.05, 0.24)	0.20	0.93	0.27	(0.40, 1.47)	p<0.01
Age of the first sexual intercourse, ≥16 years old (vs. <16 years old)	0.02	0.11	(-0.19, 0.23)	0.84	-1.74	0.38	(-2.49, -1.00)	p<0.01
Age	0.02	0.02	(-0.02, 0.06)	0.35	0.40	0.08	(0.24, 0.55)	p<0.01
Gender, Female (vs. Male)	-0.06	0.07	(-0.20, 0.08)	0.37	-0.08	0.26	(-0.59, 0.43)	0.76
Sexual orientation, Bisexual/Homosexual (vs. Heterosexual)	0.06	0.08	(-0.09, 0.21)	0.41	0.30	0.27	(-0.24, 0.84)	0.27
Relationship status, Currently in a relationship (vs. Not currently in a relationship)	0.41	0.08	(0.25, 0.57)	p<0.01	0.69	0.30	(0.10, 1.28)	p<0.05
Monthly income, ≥HKD 5,000 (vs. <HKD5,000)	0.27	0.08	(0.11, 0.43)	p<0.01	1.34	0.30	(0.75, 1.93)	p<0.01
Smoking status, Current smoker (vs. Non current smoker)	0.02	0.12	(-0.22, 0.25)	0.88	1.52	0.43	(0.68, 2.36)	p<0.01
Drinking status, Current drinker (vs. Non current drinker)	-0.02	0.07	(-0.17, 0.12)	0.74	0.70	0.27	(0.18, 1.23)	p<0.01
R-square	16.64%				47.46%			

All variance inflation factors < 2.

*p-value<0.05 by independent t-test

**p-value<0.01 by independent t-test

Besides, we found no interaction effects “between use of dating apps and gender” and “between use of dating apps and sexual orientation” (data not shown).

To further explore the association between length of time using dating apps and number of sexual partners in the last 3 months and in the last 1 month, the length of time using dating apps was put into the regression model. Subjects using dating apps for more than 12 months (relative to nonusers) were associated with a higher number of sexual partners in the last 3 months and last 1 month. The results are shown in Table 5.

**Table 5:** Factors associated with the number of sexual partners by multiple linear regressions (length of time using dating apps).

Independent variable	Number of sexual partners in the last 3 months (n = 244)				Number of sexual partners in the last 1 month (n = 244)			
	Coeff	SE	95% CI	p-value	Coeff	SE	95% CI	p-value
Using dating apps less than 1 month (vs. nonuser)	0.17	0.29	(-0.40, 0.75)	0.56	0.10	0.24	(-0.37, 0.57)	0.67
Using dating apps 1–2 months (vs. nonuser)	-0.17	0.29	(-0.74, 0.41)	0.57	-0.23	0.24	(-0.69, 0.24)	0.33
Using dating apps 3–12 months (vs. nonuser)	0.11	0.18	(-0.24, 0.45)	0.54	-0.18	0.14	(-0.46, 0.10)	0.21
Using dating apps >12 months (vs. nonuser)	0.30	0.09	(0.11, 0.48)	p<0.01	0.16	0.08	(0.01, 0.31)	p<0.05
Age of the first sexual intercourse, ≥16 years old (vs. <16 years old)	0.03	0.13	(-0.22, 0.29)	0.79	0.02	0.10	(-0.18, 0.23)	0.82
Age	0.03	0.03	(-0.02, 0.08)	0.28	0.01	0.02	(-0.03, 0.06)	0.51
Gender, Female (vs. Male)	-0.05	0.09	(-0.22, 0.12)	0.54	-0.07	0.07	(-0.21, 0.06)	0.30
Sexual orientation, Bisexual/Homosexual (vs. Heterosexual)	0.09	0.09	(-0.09, 0.28)	0.34	0.08	0.08	(-0.07, 0.23)	0.31
Relationship status, Currently in a relationship (vs. Not currently in a relationship)	0.41	0.10	(0.22, 0.61)	p<0.01	0.41	0.08	(0.25, 0.57)	p<0.01
Monthly income, ≥HKD 5,000 (vs. <HKD5,000)	0.24	0.10	(0.04, 0.44)	p<0.05	0.24	0.08	(0.08, 0.41)	p<0.01
Smoking status, Current smoker (vs. Non current smoker)	0.17	0.15	(-0.12, 0.46)	0.24	0.01	0.12	(-0.22, 0.25)	0.91
Drinking status, Current drinker (vs. Non current drinker)	0.05	0.09	(-0.13, 0.22)	0.61	-0.01	0.07	(-0.15, 0.13)	0.87
R-square	17.58%				19.80%			

All variance inflation factors < 2.

*p-value<0.05 by independent t-test

**p-value<0.01 by independent t-test

### Factors associated with risky sexual behaviours

The results of multiple linear regression analysis found that factors associated with having unprotected sexual intercourse with more lifetime sexual partners included being a user of dating apps, having one’s first sexual intercourse before 16 years of age, being older, currently being in a relationship, having a monthly income greater than HKD$5,000, being a current smoker, and being a current drinker. The results are shown in Table 4. The results of multiple logistic regression found that users of dating apps (aOR: 0.52) and current drinkers (aOR: 0.40) were less likely to have consistent condom use. The results are shown in Table 6.

**Table 6:** Correlates of consistent condom use by multiple logistic regressions.

		Overall	Condom use		p-value^	Multiple logistic regression (n = 247)	
			Consistent (100%)	Inconsistent		Adjusted OR (95% CI)	p-value
**Use of dating apps, n (%)**					p<0.01		p<0.05
	No	90 (34.62)	38 (51.35)	52 (27.96)		1.00	
	Yes	170 (65.38)	36 (48.65)	134 (72.04)		**0.52 (0.28–0.97)**	** **
Age of the first sexual intercourse						** **	0.59
	<16 years	37 (14.23)	5 (6.76)	32 (17.20)	p<0.05	1.00	** **
	≥16 years old	223 (85.77)	69 (93.24)	154 (82.80)		1.34 (0.46–3.95)	** **
Mean age (SD)**					p<0.01		0.05
** **		20.54 (1.66)	20.08 (1.37)	20.73 (1.73)		0.81 (0.66–1.00)	** **
**Gender, n (%)**					p<0.05		0.81
	Male	134 (52.34)	30 (41.67)	104 (56.52)		1.00	
	Female	122 (47.66)	42 (58.33)	80 (43.48)		1.08 (0.57–2.03)	
**Sexual orientation, n (%)**					p<0.05		0.16
	Heterosexual	180 (69.77)	59 (80.82)	121 (65.41)		1.00	
	Bisexual/homosexual	78 (30.23)	14 (19.18)	64 (34.59)		0.59 (0.28–1.23)	** **
**Relationship status, n (%)**					0.26		0.65
	Not currently in a relationship	62 (23.85)	14 (18.92)	48 (25.81)		1.00	
	Currently in a relationship	198 (76.15)	60 (81.08)	138 (74.19)		0.84 (0.39–1.80)	** **
**Monthly income, n (%)**					p<0.01		0.13
	<HKD5,000	184 (72.73)	62 (86.11)	122 (67.40)		1.00	
	≥HKD 5,000	69 (27.27)	10 (13.89)	59 (32.60)		0.52 (0.23–1.21)	
**Smoking habit, n (%)**					0.06		0.60
	Non- or ex-smoker	234 (90.35)	71 (95.95)	163 (88.11)		1.00	
	Current smoker	25 (9.65)	3 (4.05)	22 (11.89)		0.70 (0.18–2.69)	** **
**Drinking habit, n (%)**					p<0.01		p<0.01
	Non- or ex- drinker	98 (37.84)	42 (56.76)	56 (30.27)		1.00	
	Current drinker	161 (62.16)	32 (43.24)	129 (69.73)		**0.40 (0.21–0.76)**	** **

All variance inflation factors < 2.

Similar to the previous results, we found no interaction effects “between use of dating apps and gender” and “between use of dating apps and sexual orientation” (data not shown).

Users of dating apps (aOR: 1.93) and bisexual or homosexual subjects (aOR: 2.57) were more likely not to have used condoms the last time they had sexual intercourse. Moreover, female subjects were more likely to report that she or her partner did not use a condom the last time she had sexual intercourse (aOR: 2.00). The results are shown in Table 7. Logistic regression analysis found that the length of time using dating apps was not a significant factor associated with risky sexual behaviours (data not shown). Similar to the previous results, we found no interaction effects “between use of dating apps and gender” and “between use of dating apps and sexual orientation” (data not shown).

**Table 7:** Correlates of condom use at the last sexual intercourse by multiple logistic regressions.

		Overall	Condom use at the last sexual intercourse		p-value^	Multiple logistic regression (n = 246)	
			No	Yes		Adjusted OR (95% CI)	p-value
**Use of dating apps, n (%)**					p<0.01		p<0.05
	No	89 (34.50)	28 (22.40)	61 (45.86)		1.00	
	Yes	169 (65.50)	97 (77.60)	72 (54.14)		**1.93 (1.04–3.59)**	** **
**Age of the first sexual intercourse**							0.81
	<16 years	37 (14.34)	24 (19.20)	13 (9.77)	<0.05		
	≥16 years old	221 (85.66)	101 (80.80)	120 (90.23)		0.90 (0.38–2.14)	** **
**Mean age (SD)**					p<0.01		0.07
** **		20.54 (1.66)	20.25 (1.50)	20.85 (1.77)		1.18 (0.99–1.42)	** **
**Gender, n (%)**					0.71		p<0.05
	Male	133 (52.16)	63 (50.81)	70 (53.44)		1.00	
	Female	122 (47.84)	61 (49.19)	61 (46.56)		**2.00 (1.08–3.69)**	
**Sexual orientation, n (%)**					p<0.01		p<0.01
	Heterosexual	179 (69.65)	71 (56.80)	108 (81.82)		1.00	
	Bisexual/homosexual	78 (30.35)	54 (43.20)	24 (18.18)		**2.57 (1.36–4.86)**	** **
**Relationship status, n (%)**					0.24		0.94
	Not currently in a relationship	61 (23.64)	34 (27.20)	27 (20.30)		1.00	
	Currently in a relationship	197 (76.36)	91 (72.80)	106 (79.70)		0.97 (0.48–1.98)	** **
**Monthly income, n (%)**					p<0.01		0.25
	<HKD5,000	183 (72.91)	76 (63.33)	107 (81.68)		1.00	
	≥HKD 5,000	68 (27.09)	44 (36.67)	24 (18.32)		1.51 (0.75–3.06)	
**Smoking habit, n (%)**					0.06		0.42
	Non- or ex-smoker	232 (90.27)	107 (86.29)	125 (93.98)		1.00	
	Current smoker	25 (9.73)	17 (13.71)	8 (6.02)		1.53 (0.54–4.30)	** **
**Drinking habit, n (%)**					p<0.05		0.08
	Non- or ex- drinker	98 (38.13)	38 (30.65)	60 (45.11)		1.00	
	Current drinker	159 (61.87)	86 (69.35)	73 (54.89)		1.71 (0.93–3.14)	
**Type of last sexual partner, n (%)**					p<0.01		0.07
	Committed partner (boyfriend/girlfriend)	203 (78.68)	84 (67.20)	119 (89.47)		1.00	
	Casual partner	55 (21.32)	41 (32.80)	14 (10.53)		2.08 (0.93–4.67)	

Model Chi-square = 48.897, df = 10, p < 0.001; Nagelkerke R-square = 0.240; Hosmer & Lemeshow Test, Chi-square = 9.666, df = 8, p = 0.289.

All variance inflation factors < 2.

## Discussion

To the best of our knowledge, this was the first study to examine the association between the use of dating apps and sexual health in a sample of heterosexual, bisexual and homosexual subjects. The present study found that the use of dating apps was associated with having more sexual partners, having unprotected sexual intercourse with more sexual partners, an increased likelihood of having inconsistent condom use and an increased likelihood of not having used a condom the last time the subject had sexual intercourse. It appeared that dating apps tended to skew their users toward risky sexual encounters.

More than half of the study sample used dating apps suggesting that dating apps are popular among college students. After controlling for sociodemographic factors, users of dating apps had 87% more likely to have sexual intercourse experience than nonusers. There are at least two possible explanations. First, the nature of dating apps with their convenience, accessibility and mobility can facilitate sexual encounters [19]. Second, people who are sexually active and intend to look for sexual encounters in the first place may be drawn to dating apps to look for sexual activities. This merits further investigation to understand the causal relationship between using dating apps and the initiation of sexual intercourse.

### Multiple sexual partners

Compared with nonusers, dating app users were more likely to have more sexual partners in their entire lives (4.07 vs. 1.72) and in the last 3 months (1.12 vs. 0.81). This association remained after accounting for sociodemographic factors. A previous study on homosexual men also found that, compared with nonusers, app users reported significantly more sexual partners in their entire lives and in the last 3 months [8]. Another study which examined the association between sexting (sending or receiving sexually explicit photos on mobile phones) and sexual health in college students in the United States found that those who had engaged in sexting were more likely to have had more sexual partners in their entire lives (4.73 vs. 2.41) and in the last 3 months (1.22 vs. 0.79) than those who had not [18]. Having multiple sexual partners is problematic because numerous studies suggest that it is associated with sexually transmitted disease infection, recreational drug use, alcohol consumption, dating violence and unplanned pregnancy [7, 20–22].

We also found that, compared with nonusers, those who used dating apps for more than 12 months were more likely to have more sexual partners in the last 3 months and in the last 1 month. Conversely, there was no significant difference in the number of sexual partners in the last 3 months and the last 1 month between nonusers and those who used dating apps less than 12 months. It appeared that using dating apps can lead to an increase in the number of sexual partners. There were several possible explanations. First, a previous qualitative study about online dating suggested that online dating websites can extend the number of people users meet and engage with sexually [1]. Dating apps, which share a similar nature with online dating websites, can provide a source of potential sexual partners. Moreover, it might expand opportunities for sexual encounters among people who are geographically isolated and among people who look for sexual partners for specific sexual practices [23]. Second, nowadays people carry their smartphones with them at all times. Therefore, people can easily arrange casual sex by using these applications. Third, it was suggested that people were more comfortable and ready to talk about sex in an online environment [1, 24].

The association between early sexual initiation and the number of sexual partners is consistent with that found in the United States [25] and mainland China [26] and is not unique to the population in the present study. Contrary to previous studies which found that sexual minority youths were more likely to have multiple sexual partners in their entire lives and in the last 3 months, this association cannot be found in the present study [7].

### Unprotected sexual intercourse

Contrary to the findings of previous studies about the use of dating apps among homosexual men [6, 8], we found that, compared with nonusers, apps users were more likely to have had unprotected sexual intercourse with more sexual partners, inconsistent condom use and no condom use when they last had sexual intercourse. However, it is hard to compare our results with those of the previous studies due to differences in study populations, control groups, the recall period of condom use and the definition of sexual intercourse. Subjects of the previous studies were all homosexual. Studies by Grosskopf et al and Lee et al compared the pattern of condom use between app users and subjects who used the Internet (not dating apps) for dating [6, 9]. It was possible that both groups had similar sexual practices, leading to no difference in the pattern of condom use. The recall period of condom use in the previous studies was the last 3 months [6, 8, 16]. The previous studies only assessed unprotected anal intercourse whereas the present study examined condom use in anal, oral and virginal intercourse. Even though the risk of HIV transmission through oral sex is much lower than anal or vaginal sex, other viral and nonviral sexually transmitted diseases such as gonorrhoea, herpes and syphilis can be transmitted through oral sex [27, 28]. The National Health Service of the United Kingdom and the Centers for Disease Control and Prevention in the United States also suggest that using a condom can make oral sex safer. Conversely, it was found that college students in the US who engaged in sexting were more like to have had more total unprotected vaginal and anal sex acts in the last 3 months than those who did not (7.97 vs. 2.92) [18]. A previous study on heterosexual women found that 77% of women who met an Internet partner for a sexual encounter did not use a condom [29]. Several studies on homosexual men also suggested that seeking sexual partners online was associated with inconsistent condom use and unprotected anal intercourse [30, 31].

### Clinical implications and recommendations

First, given the popularity of dating apps and smartphones nowadays, it is impractical and unrealistic to stop people using dating apps. If using dating app causes more risky sexual behaviours, clinicians should develop interventions to promote the safe use of dating apps in order to reduce the likelihood of engaging in risky sexual behaviours among app users, especially for those with no history of risky sexual behaviours. Second, we found that using dating apps was associated with more risk sexual behaviours. Therefore, users of dating apps should be targeted in risk assessment, screening as well as risk stratification. Third, the developers of these dating apps should take social responsibility. Educational elements such as popup preventive messages and notification to promote safe sex practices should be incorporated into the apps [32]. Besides, school-based interventions to promote safe sex and availability of condoms in school campuses and dormitories should be advocated. In addition to app users, bisexual/homosexual people, women, older people and people who had their first sexual intercourse before 16 years of age should deserve more attention because they appear to engage in more risky sexual behaviours.

### Limitations

There were a number of limitations to this study. First, subjects of this study were recruited in universities by convenience sampling and thus the study findings might not be generalizable to other populations. Compared with the general populations, the age range of our study subjects were narrow and the differences in socio-demographic factors between subjects were low. However, we used multicenter survey to expand the diversities of participants from different geographic locations, study disciplines, academic performance and socioeconomic status. To establish a robust association between the use of dating apps and sexual health, people in general population should be recruited by probability sampling in future studies. Second, all outcomes were self-reported, which might have bias. However, using self-report measures to obtain data is a common and practical methodology in studies of behavioural health. To strengthen the validity and reliability of our data, all the questions were adapted from previous research studies. In addition, to avoid social desirability bias, the questionnaire was anonymous and self-completed by interviewees themselves. Third, this cross-sectional study only provided clues regarding associations, and longitudinal studies are needed to establish any causal relationship between independent variables and sexual health. One suggested alternative is to divide groups of our study subjects by a “proxy variable” that theoretically has associations with the use of dating apps but no association with risky sexual behaviours, and to further analyse if this “proxy variable” has a statistically significant association with the risky sexual behaviours. However, we did not have such variables in this present study. Fourth, we combined homosexual group with bisexual group in data analysis in order to improve the model fit of the regression models. Further study should recruit more people in each group and conduct subgroup analysis to explore whether there are differences in sexual risk behaviours between heterosexual, homosexual and bisexual people. Fifth, nowadays, smartphone apps are versatile. Some messenger apps also have a function for making new friends nearby. However, these apps which are not primarily for dating were not considered in the present study. Further studies might include this kind of apps to see if there are any impacts on sexual risk behaviours.

## Conclusion

The present study found a robust association between using dating apps and sexual risk behaviours including having more sexual partners, having had unprotected sexual intercourse with more sexual partners in a lifetime, inconsistent condom use in a lifetime and no condom use the last time subjects had sexual intercourse in our sample of college students, suggesting that app users had greater sexual risks. With the growing popularity of using smartphone dating apps, it is time to consider development and testing of novel interventions that can target app users so that they can stay safe when seeking sexual partners through dating apps. Further studies should also be conducted to understand the motivations for using dating apps and attempt to reveal mechanisms that may explain the relationship between using dating apps and the associated sexual risks.

## Supporting Information

S1 Instrument Study instument for assessing the use of dating apps, sexual behaviorus and sociodemograhpics. (PDF)
