# Supplementary material for: The Impacts of Using Smartphone Dating Applications on Sexual Risk Behaviours in College Students in Hong Kong
Source: PLoS One. 2016 Nov 9;11(11):e0165394. doi: 10.1371/journal.pone.0165394 (PMC5102411; doi:10.1371/journal.pone.0165394)
Supplement: S1 Instrument — (PDF) [file pone.0165394.s001.pdf]

S1 Instrument

Use of dating apps

- Do you use dating applications (apps)?
  Yes/ No

- How long have you started using dating app(s)?
  < 1 month/ 1-2 months/ 3-12 months/ >12 months

Sexual behaviours

- Have you ever had sexual intercourse?
  Yes/ No

- *Have you done any sexual touching?*
  Yes with girls/ Yes with boys/ Yes with both boys and girls/ No

- How old were you when you had sexual intercourse for the first time?
  Age:

- During your life, with how many people have you had sexual intercourse?
  Number:

- During the past 3 months, with how many people did you have sexual intercourse?
  Number:

- During the past 1 month, with how many people did you have sexual intercourse?
  Number:

- During your life, with how many people have you had unprotected sexual intercourse?
  Number:

- During your life, how often do you/ your partner(s) use condom when you have sexual intercourse?
  Never/ Sometimes (less than half of the occasions)/ Half of the occasions/ Frequently (more than half of the occasions)/ Always (100%)

- The last time you had sexual intercourse, did you or your partner use a condom?
  Yes/ No

Sociodemographics

- Age

- Gender
  Male/ Female

- Sexual orientation

  Attracted to the opposite sex/ Attracted to both the opposite and the same sex/ Attracted to the same sex

- Relationship status
  Currently in a relationship/ Not Currently in a relationship

- Occupational status
  Students/ Others (employed, self-employed, employer, seeking job, retired)

- Individual monthly income
  <$5,000/ ≥$5,000

- Do you smoke?
  Yes/ No

- Do you drink?
  Yes/ No
